# Novel variants of *SYNGAP1* associated epileptic encephalopathy: two cases report and literature review

**DOI:** 10.1186/s42494-022-00114-z

**Published:** 2023-02-21

**Authors:** Xingying Zeng, Yong Chen, Xiongying Yu, Yuanyuan Che, Hui Chen, Zhaoshi Yi, Jie Qin, Jianmin Zhong

**Affiliations:** 1 Molecular Diagnostic Laboratory, Children’s Hospital of Jiangxi Province, Nanchang, 330006 China; 2 Department of Neurology, Children’s Hospital of Jiangxi Province, Nanchang, 330006 China

**Keywords:** *SYNGAP1*, Epilepsy, Prednisone, Clonazepam, Case report

## Abstract

**Background:**

*SYNGAP1* is a significant genetic risk factor for global developmental delay, autism spectrum disorder, and epileptic encephalopathy. De novo loss-of-function variants in this gene cause a neurodevelopmental disorder, for example, early-onset and drug-refractory seizures. We report two children with global developmental delay and epileptic encephalopathy, which are caused by *SYNGAP1* gene novel mutations, and drug treatment is effective.

**Case presentation:**

We report a boy and a girl presented with global developmental delay when they were young babies; as they grew up, cognitive impairment and social-communication disorder became more and more prominent; unfortunately, the patients developed into various seizure types, including eyelid myoclonia, myoclonic and absences when the boy was 1 year 8 mouths old and the girl was 3 years old. The two patients were found two previously unknown mutations by high throughput sequencing [c.3271_ c.3272insT; (p.L1091L fs*62), c.2515A > T (p.K839*)] in exon 15 of the *SYNGAP* in the proband. Sanger sequencing confirmed the heterozygous nature, and neither of their parents carried the same mutation. The girl treated with valproic acid and prednisone became seizure-free, and valproic acid and levetiracetam combined with clonazepam were influential in the other.

**Conclusions:**

The global developmental delay and epileptic encephalopathy of the children were probably due to the pathogenic mutation of the *SYNGAP1* gene, and prednisone and clonazepam may be effective in achieving seizure-free.

## Background

*SYNGAP1* (Gene ID: 8831 /OMIM: 603,384, Synonyms *SYNGAP*, *RASA5,* and *KIAA1938*) is located in the 6p21.3 chromosome. It encodes SynGAP protein, a Ras-GTPase activating protein that plays an important role in synaptic signal transmission [1, 2]. Heterozygous loss-of-function variants in *SYNGAP1* cause autosomal mental retardation type 5 (MRD5; phenotype MIM 612621; gene/locus MIM 603384), and its common phenotypes include cognitive impairment, speech disorder, behavioral deficits, and epilepsy [3]. The loss-of-function variants of *SYNGAP1* are surprisingly common, with the incidence reported as 1–4/10,000 individuals, or approximately 0.5–4.0% of intellectual disability (ID) cases, making it one of the most common causes of ID with epilepsy [3, 4]. As *SYNGAP1* understanding and research progressed, people found more and more the diversity of disease range and genetic variation related to *SYNGAP1* pathogenicity. Patients with pathogenic *SYNGAP1* variants were called *SYNGAP1*-related encephalopathy [3].

Although there are many reports about *SYNGAP1*-related encephalopathy in the medical community [2, 5, 6], we report that two children with ID and epilepsy carry the pathogenic variation of the *SYNGAP1* gene, and the interpretation of this locus has not been registered. Now the cases are shared, which enriches the heritage variation database and increases the choice of treatment options.

## Case presentation

### Case 1

**Fig. 1:**
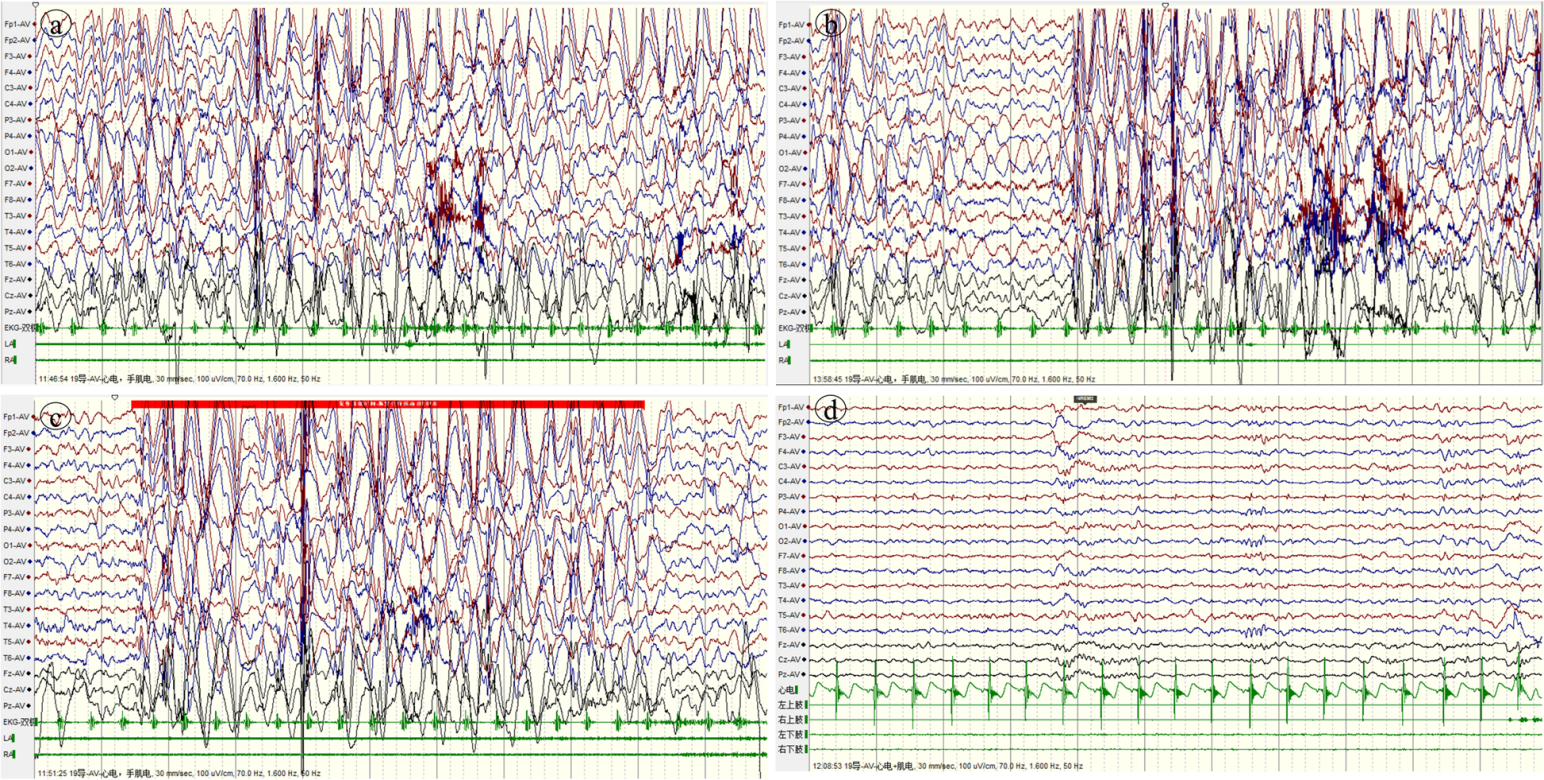
(EEG results of case 1): **a**. Before treatment, interictal sleep VEEG showed that in seizure interval there were extensive 1.5-2.5 Hz, high amplitude slow spike slow waves and focal spinous slow waves in occipital or prefrontal region; **b-c**. Epileptic discharges could be seen in the seizure period before treated, considering eyelid myoclonus and atypical absence seizures (In the picture b, the child sitting on the bed with her head slightly tilted back and her eyes blinking; in the picture c, the child sitting on the bed with her eyes looking forward without obvious movement); **d**. After treatment, EEG during sleep proved no obvious epileptiform discharge. Electromyogram: **a,b,c** (on both deltoid muscles) & **d** (on both deltoid muscles and both quadriceps femoris muscles)

**Fig. 2:**
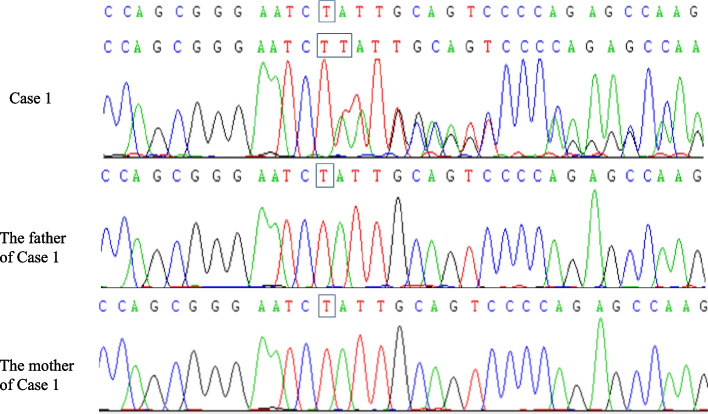
Sanger sequencing results of case 1. A denovo heterozygous frameshift mutation in the *SYNGAP1* gene: c.3271(exon15)_c.3271(exon15) insT (p.leu1091Leufs*62) (NM_006772) in the proband of case1, and the mutation was not found in the her parents

**Fig. 3:**
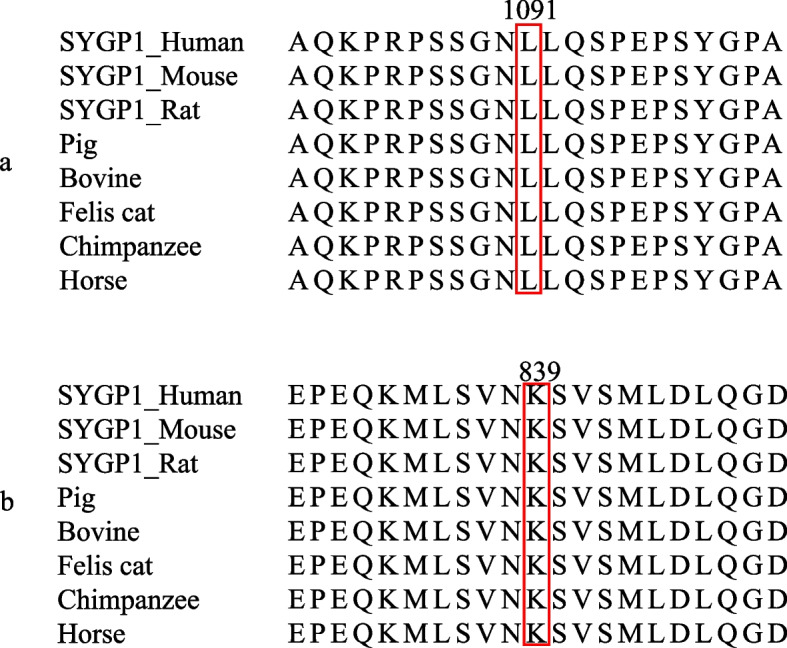
Conservative analysis. Fig.3-**a** shows that the ectopic site of case1 is highly conserved in different species. Fig.3-**b** shows that the ectopic site of case 2 is highly conserved in different species

A 3-year-old Chinese girl of Han nationality, who expressed global developmental delay since childhood. She could look up at age of 6 months, walk independently at age of 33 months and only unconsciously shout "Mom and Dad" at age of 3. Her parents are not related, and her mother naturally conceived, had full-term childbirth, and had no history of hypoxia and asphyxia. At the age of 31 months, the girl suddenly appeared to have seizures in the form of a head tilted back and occurred in clusters, each for 3–4 s, several times a day.. Sometimes she would stop suddenly and shake her body slightly when walking; occasionally, she nodded forward. Video electroencephalogram (VEEG) showed that seizure intervals were extensive 1.5–2.5 Hz, high amplitude slow spike slow waves, and focal spinous slow waves in the occipital or prefrontal region. Epileptic discharges can be seen in the seizure period, considering myoclonic seizures and atypical absence seizures (Fig. 1-a,b,c). MRI of the head showed no obvious abnormality. The Denver Developmental Screening Test (DDST) of this little girl suggests that she has global developmental abnormalities (thick motor development was equivalent to 13 months, fine motor adaptability was equal to 11 months, personal social development level was equivalent to 10 months, and language ability was equivalent to 10 months). High throughput sequencing (HTS) results showed *SYNGAP1*: c.3271 (exon15)_ c. 3272 (exon15) InsT, p.L1091L FS * 62. Sanger sequencing showed that the child carried *SYNGAP1* gene c.3271_ c.3272 InsT heterozygous variation, and her parents were wild-type (Fig. 2). The GnomAD and HGMD database did not include this ectopic variant site (the last check was on May 18, 2022). The Uniport evaluated the ectopic variant site located in a highly conserved region (Fig. 3-a) and classified it as a "pathogenic" variant according to the ACMG variation classification standard (PVS1 + PS2 + PM2). Finally, the little girl was diagnosed with *SYNGAP1*-related encephalopathy. " valproic acid (VPA), 10 mg/kg/d" treatment was initially given. The number of seizures was decreased for a while after she took medicine. After the dosage was increased to 15 mg/kg/d, the number of seizures was increased. The child had a variety of attack forms, the EEG discharge index exceeded 70%, the mental retardation was obvious, and even there were signs of regression. We combined the *SYNGAP1*-related encephalopathy, often complicated with refractory epilepsy and epileptic encephalopathy, so we gave the girl high-dose prednisone (60 mg/d). The seizure disappeared on the third day, and the EEG was normal after 2 weeks. The EEG was supplemented, as shown in Fig. 1-d. After that, prednisone was gradually reduced and stopped within 2 months. There was no seizure after follow-up for 1 year.

### Case 2

**Fig. 4:**
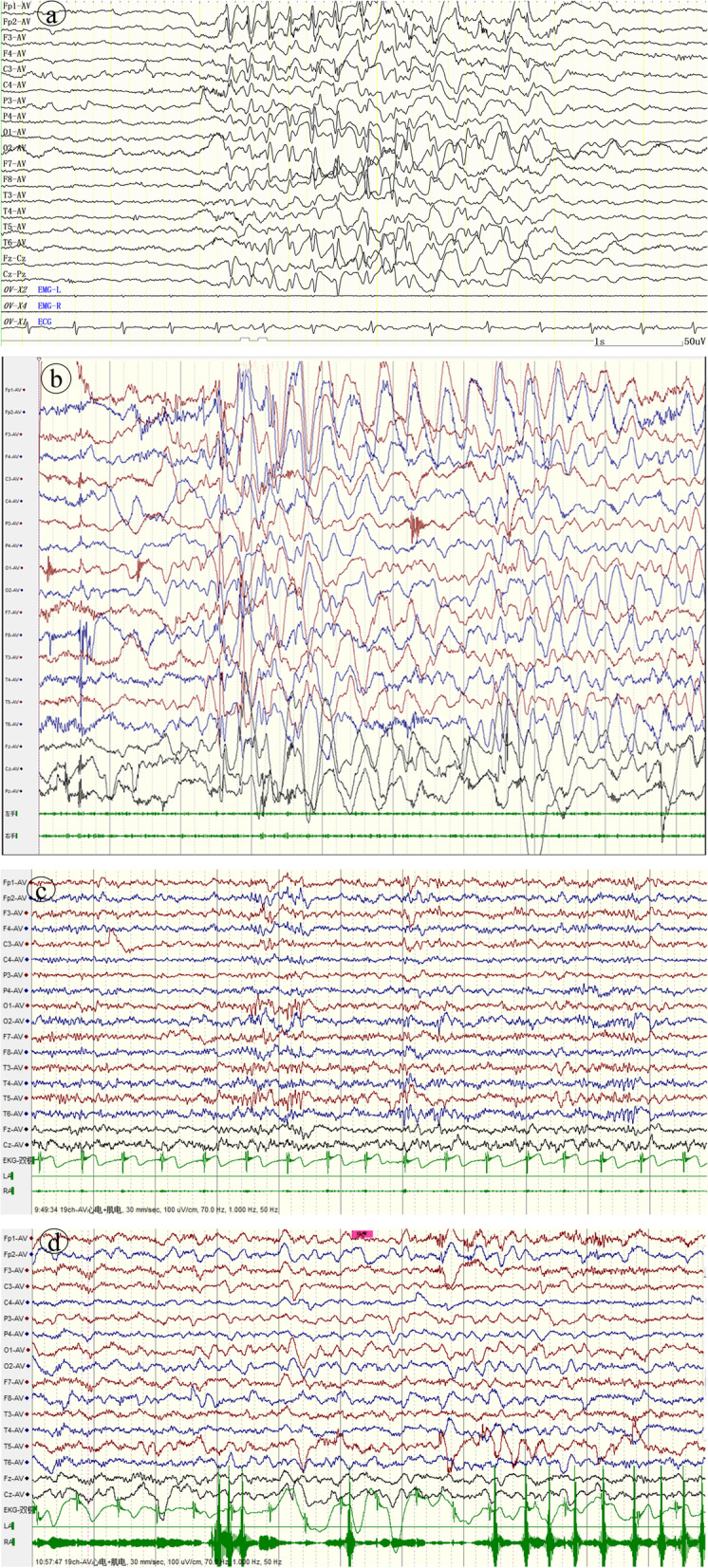
(EEG results of case 2): **a **Before treatment, spikes and slow waves in all regions of the whole brain could be seen in the EEG during the attack period; **b **Before treatment, fast rhythm or multi spike slow wave intermittent burst could be seen in EEG during seizure interval and awake period; **c-d **There was no obvious epileptiform discharge in EEG during sleep and awake after treatment. Electromyogram: on both deltoid muscles

**Fig. 5:**
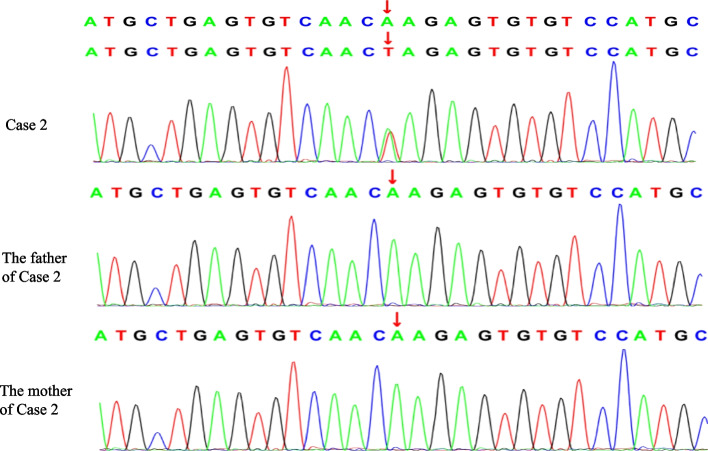
Sanger sequencing results of case 2. A denovo heterozygous missense mutation in the *SYNGAP1* gene: c.2515(exon15) A > T (p.Lys839stop, 505) (NM_006772) in the proband of case 2, and themutation was not found in his parents

A boy was 3 years and 7 months old, and his motor language development was backward since birth; he looked up at age of 5–6 months, could walk independently at the age of 1 year and 6 months, and still couldn't speak at the age of 3 years and 7 months. His parents are not related, and his mother conceived naturally, gave birth naturally at full term, and had no history of hypoxia asphyxia. At 1 year and 7 months, the child was diagnosed with "autism spectrum disorder (ASD)" due to backward language development and went to the hospital. When he was 1 year and 10 months old, he had paroxysmal eyes upturning and blinking, with sluggish response and remission for several seconds, sometimes accompanied by head lowering. VEEG showed that spikes and slow waves were paroxysmal in all regions of the whole brain during an attack, and fast rhythm or multi spikes and slow waves were intermittent in the whole brain during sleep (Fig. 4-a,b). We considered the seizure type as eyelid myoclonus, atonic seizure, and absence. MRI of the head showed no obvious abnormality. HTS results revealed *SYNGAP1*: c.2515 (exon15) A > T, p.K839*. Sanger sequencing showed that the child carried a heterozygous variant of *SYNGAP1* c.2515 (exon15) A > T, and his parents were wild-type (Fig. 5). The GnomAD and HGMD database did not include the ectopic variant site (the last check was on May 18,2022). Some software predicted that this variant could terminate the amino acid translation in advance. The Uniport website evaluated that this ectopic variant site was located in a highly conserved region (Fig. 3-b) and was classified as a "pathogenic" variant according to the Annotation ACMG variation classification standard (PVS1 + PS2 + PM2 + PP3). *SYNGAP1*-related encephalopathy was diagnosed. After he took valproic acid, the attack frequency decreased slightly, eating could induce eyes upturning, and this situation still existed until 40 mg/kg/d. The number of seizures decreased after adding clonazepam at 2 years and 8 months. When clonazepam was added to 0.1 mg/kg/d, gait instability appeared, and his father refused to increase the dosage of clonazepam. Therefore, levetiracetam was added gradually to 35 mg/kg/d, but there was still an attack. Combined with the previous treatment of clonazepam, the curative effect of clonazepam was good, and the doctor added the child's clonazepam to 0.15 mg/kg/d. There was no obvious abnormality in EEG after 1 month (Fig. 4-c,d). The EEG was abnormal again after 3 months of follow-up. However, there was no obvious attack. Then added clonazepam to 0.2 mg/kg/d, the boy’s EEG was normal after 3 months.

## Discussion

Case 1 had absence, eyelid myoclonus, and nodding, but our VEEG proved that her nodding was a non-epileptic seizure. The child did not have a tonic seizure and followed partial seizures. At present, epilepsy syndrome cannot be classified [7]. Case 2 had eyelid myoclonus, atonic attacks, and absence, accompanied with seizures induced by eating. Stülpnagel found that epilepsy associated with *SYNGAP1* mutation had the characteristics of food induction and eye closure sensitivity [6]. When simply looking up the *SYNGAP1*-related diseases in the Orphanet database, we would find more than 80% of the patients have similar symptoms such as abnormal pain, speech and language development delay, generalized seizures, global development delay, and intellectual disability. The top three categories of phenotypes present in patients with sequence variants are global development delay, delayed speech, language development, and seizure in the Decipher database. The two children reported in this paper had extended developmental delay and intellectual disability since childhood, and the disease seriously impaired their language ability. They all had seizures with multiple seizure forms, highly similar to the clinical phenotypes of previously reported cases.

*SYNGAP1* is included in the developing disorder genes database and is the causative gene of intelligent developing disorder autosomal dominant type 5. The GnomAD PLI value is 1.0, which is sufficient evidence for haploinsufficiency. Among patients with an ACMG rating of P / LP, 39 / 50 of the variants included are frameshift and nonsense variants caused by small insertions and deletions in the Decipher database. At the same time, the Decipher database found that the highest phenotype frequency was global development delay in patients with sequence variants. In contrast, the phenotype was autistic behaviors in the patients with copy number variants (https://www.deciphergenomics.org). There were 205 patients rated P in the Clinvar database, of which 160/205 were frameshift and nonsense variants caused by small insertions and deletions (https://www.ncbi.nlm.nih.gov/clinvar). These data all support that the variant type described in this paper is pathogenic. After the Vercards Web, c.2515A > T was predicted to be harmful by multiple software, including mutation, CADD, Dann, fathmm_MKL, Eigen, and Genocanyons. Unfortunately, there was no Software prediction c.3271_c. 3272insT (http://159.226.67.237/sun/varcards). Combined with the database data, variant sources, and variant types, the two variant sites reported in this paper are supported as newly discovered pathogenic mutation sites.

The *SYNGAP1* variation in two children reported by us appears in exon 15, which shows developmental encephalopathy with epilepsy. Developmental encephalopathy belongs to a severe phenotype of *SYNGAP1*-related encephalopathy. It is said that the clinical manifestations of *SYNGAP1* encephalopathy patients with mutations in exons 5 to 19 are heavier than those in exons 1–4, and the prognosis of drug treatment is worse [2, 5]. We can understand this result by binding the SynGAP protein structure. SynGAP protein consists of N-terminal, C-terminal, and core region. The core region composed of C2, GAP, and SH3 is unchanged, and the N-terminal and C-terminal at both ends contain variable domains. At present, five N-terminals (A1, A2, B, C, and D) and four C-terminals (α1、α2, β, and γ) have been found, and they constitute 20 subtypes [1, 8–10]. The variation of exons 1–4 will only affect the SynGAP-A subtype and will not affect other subtypes, while the amino acids encoded by exons 5–16 correspond to the fixed core region, which will affect SynGAP proteins of all subtypes, so the phenotype is relatively heavier.

Literature found that the mutations of *SYNGAP1*-related encephalopathy are all new mutations [2], and the case reported in this paper is also a new variant. De novo is the most common cause of ID, indicating that the genes causing ID are mostly single allele diseases. For the *SYNGAP1*, *SYNGAP1*^−/+^ mouse model can appear with clinical phenotype, and homozygous mutation will cause embryonic death; On the other hand, it is difficult for patients with ID and ASD to get married and have children, which reduces the probability of inheritance of pathogenic variation [1].

It is reported in the literature that *SYNGAP1*-related encephalopathy is refractory, and usually, two or even six epilepsy drugs are used in combination. Valproic acid and lamotrigine are the most commonly used drugs. Polytherapy combinations resulting in seizure freedom included levetiracetam and topiramate, valproate and lamotrigine, and cannabidiol, valproate, with lamotrigine [3]. Although the literature reported that the prognosis of patients with exon 5 to exon 19 mutation was poor [2, 5], the therapeutic effect of our two children was acceptable. Our cases have various seizure forms, so the first choice is sodium valproate, a broad-spectrum antiepileptic drug, which does have the effect of reducing seizures. The child in case 1 finally controlled the seizure after adding a hefty prednisone dose, and the epileptic discharge disappeared. In the previous literature [2], a child with West syndrome caused by *SYNGAP1* mutation controlled the attack after using ACTH alone. The girlin this report doesn't have West syndrome, but the curative effect is excellent after using prednisone, which provides an idea for our future treatment. Although the child in case 2 was treated with valproic acid, levetiracetam, and clonazepam, combined with their medical history, we could find that clonazepam should be the most effective drug. However, why is clonazepam effective? The reason is found in the pathogenic mechanism of *SYNGAP1* mutation.

SynGAP protein is a brain-specific synaptic RasGTP activator protein 1, mainly expressed in the dendritic spines of cortical vertebral neurons, and plays a vital role in synaptic signal transmission [11]. The C-terminal of SynGAP protein has a CC domain, which can bind to PDZ-containing proteins such as PSD95 [11]. Postsynaptic density protein (PSD) is a disc-shaped structure on the cytoplasmic side of the postsynaptic membrane of glutamate excitatory synapses in the mammalian central nervous system, which forms a complex with NMDAR and other substances. Under unstimulated baseline conditions, SynGAP was mainly located in the core region of PSD within 40 nm of the postsynaptic plasma membrane [12]. After depolarization, SynGAP is translocated from the core region of PSD to the external cytoplasmic part of the PSD complex [12]. Based on this feature, Walkup put forward the "slot" hypothesis. SynGAP occupies the slot containing PDZ protein in PSD [13]. When SynGAP in synapse decreases, more PDZ binding "sites" can be obtained. Other proteins will occupy these sites with the PDZ binding domain. Some of these proteins can stabilize and recruit AMPAR at a synapse, resulting in increased synaptic strength and imbalance of cell excitability. We all know that the imbalance of synaptic excitability (E) and inhibitory (I) balance is the basis of the pathogenesis of various central nervous system diseases [14]. If synaptic E/I balance tends to be excited, it will cause epilepsy [14]. Clonazepam is a drug to inhibit excitation, so it can be considered to add Clonazepam to *SYNGAP1*-related encephalopathy combined with drug-refractory epilepsy.

Since E/I imbalance is often associated with many neurological diseases, such as epilepsy, mental retardation, and autism, this explains the clinical characteristics of patients with *SYNGAP1*-related encephalopathy [15]. Because some patients are in a highly excited and irritable state, in addition to epilepsy and underdevelopment, there will be many behavioral problems, including ASD、ADHD、extreme risk-taking behaviors such as climbing and jumping from a height, as well as behavioral problems such as aggression, self-injury and temper tantrums, elevated pain threshold, difficulty eating and difficulty falling asleep, which perplex the guardians [14].

## Conclusions

In summary, we reported two epilepsy children with severe intellectual impairment and global growth retardation. HTS showed that the *SYNGAP1* gene was mutation in the two children, respectively c.3271_ c.3272insT (p.L1091L fs*62) and c.2515A > T (p.K839*). Variants were verified by first-generation sequencing. This locus was a new variant in children, and there was no relevant report on HGMD. Through clinical characteristics and pathogenicity evaluation, this variant was pathogenic. These two children had multiple seizure forms and many EEG discharges. One case was treated with sodium valproate combined with prednisone, and the other was treated with sodium valproate and levetiracetam combined with clonazepam. Their treatment was effective, which provided a treatment idea for the epilepsy of *SYNGAP1*-related encephalopathy in the future.

## Data Availability

The datasets used and analyzed during the current study are available from the corresponding author upon reasonable request.

## Abbreviations

ACMG: The American College of Medical Genetics and Genomics
ACTH: Adrenocorticotropic hormone
ADHD: Attention-deficit hyperactivity disorder
ASD: Autism spectrum disorder
DDST: The Denver Developmental Screening Test
HGMD: The Human Gene Mutation Database
HTS: High throughput sequencing
ID: Intellectual disability
MRI: Magnetic resonance imaging
NMDAR: N-methyl-d-aspartate receptor
PSD: Postsynaptic density protein
VEEG: Video electroencephalogram
VPA: Valproate
